# Biosynthesis of Hesperetin, Homoeriodictyol, and Homohesperetin in a Transcriptomics-Driven Engineered Strain of *Streptomyces albidoflavus*

**DOI:** 10.3390/ijms25074053

**Published:** 2024-04-05

**Authors:** Álvaro Pérez-Valero, Juan Serna-Diestro, Albert Tafur Rangel, Simona Barbuto Ferraiuolo, Chiara Schiraldi, Eduard J. Kerkhoven, Claudio J. Villar, Felipe Lombó

**Affiliations:** 1 Research Group BIONUC (Biotechnology of Nutraceuticals and Bioactive Compounds), Area of Microbiology, Department of Functional Biology, University of Oviedo, 33006 Oviedo, Principality of Asturias, Spain; apv.moratalla@gmail.com (Á.P.-V.); sernadjuan@uniovi.es (J.S.-D.); cjvg@uniovi.es (C.J.V.); 2 Instituto Universitario de Oncología del Principado de Asturias (IUOPA), 33006 Oviedo, Principality of Asturias, Spain; 3 Instituto de Investigación Sanitaria del Principado de Asturias (ISPA), 33006 Oviedo, Principality of Asturias, Spain; 4 Department of Biology and Biological Engineering, Chalmers University of Technology, SE-412 96 Gothenburg, Sweden; tafur@chalmers.se (A.T.R.); eduardk@chalmers.se (E.J.K.); 5 Novo Nordisk Foundation Center for Biosustainability, Technical University of Denmark, DK-2800 Kongens Lyngby, Denmark; 6 Section of Biotechnology and Molecular Biology, Department of Experimental Medicine, University of Campania “Luigi Vanvitelli”, Via De Crecchio 7, 80138 Naples, Italy; simona.barbutoferraiuolo@unicampania.it (S.B.F.); chiara.schiraldi@unicampania.it (C.S.); 7 SciLifeLab, Chalmers University of Technology, SE-412 96 Gothenburg, Sweden

**Keywords:** l-tyrosine feeding, flavonoid, methyltransferase, substrate flexibility

## Abstract

Flavonoids exhibit various bioactivities including anti-oxidant, anti-tumor, anti-inflammatory, and anti-viral properties. Methylated flavonoids are particularly significant due to their enhanced oral bioavailability, improved intestinal absorption, and greater stability. The heterologous production of plant flavonoids in bacterial factories involves the need for enough biosynthetic precursors to allow for high production levels. These biosynthetic precursors are malonyl-CoA and l-tyrosine. In this work, to enhance flavonoid biosynthesis in *Streptomyces albidoflavus*, we conducted a transcriptomics study for the identification of candidate genes involved in l-tyrosine catabolism. The hypothesis was that the bacterial metabolic machinery would detect an excess of this amino acid if supplemented with the conventional culture medium and would activate the genes involved in its catabolism towards energy production. Then, by inactivating those overexpressed genes (under an excess of l-tyrosine), it would be possible to increase the intracellular pools of this precursor amino acid and eventually the final flavonoid titers in this bacterial factory. The RNAseq data analysis in the *S. albidoflavus* wild-type strain highlighted the *hppD* gene encoding 4-hydroxyphenylpyruvate dioxygenase as a promising target for knock-out, exhibiting a 23.2-fold change (FC) in expression upon l-tyrosine supplementation in comparison to control cultivation conditions. The subsequent knock-out of the *hppD* gene in *S. albidoflavus* resulted in a 1.66-fold increase in the naringenin titer, indicating enhanced flavonoid biosynthesis. Leveraging the improved strain of *S. albidoflavus*, we successfully synthesized the methylated flavanones hesperetin, homoeriodictyol, and homohesperetin, achieving titers of 2.52 mg/L, 1.34 mg/L, and 0.43 mg/L, respectively. In addition, the dimethoxy flavanone homohesperetin was produced as a byproduct of the endogenous metabolism of *S. albidoflavus.* To our knowledge, this is the first time that *hppD* deletion was utilized as a strategy to augment the biosynthesis of flavonoids. Furthermore, this is the first report where hesperetin and homoeriodictyol have been synthesized from l-tyrosine as a precursor. Therefore, transcriptomics is, in this case, a successful approach for the identification of catabolism reactions affecting key precursors during flavonoid biosynthesis, allowing the generation of enhanced production strains.

## 1. Introduction

Flavonoids are bioactive phytochemicals of paramount nutraceutical, pharmaceutical, and agro-industrial importance due to the vast variety of properties they display [1,2,3]. Specifically, hesperetin, a methylated eriodictyol (at the 4′ hydroxyl position), has been reported to possess anti-inflammatory [4], neuroprotective [5,6], anti-tumor, anti-spasmodic, cardioprotective, anti-asthmatic and anti-diabetic bioactivities [7]. Another less studied eriodictyol methylated derivative, homoeriodictyol (3′-*O*-methyl eriodictyol), has been suggested to possess the capability of increasing appetite when administered with glucose in humans [8] and to increase SGLT-1 (sodium-glucose linked transporter) mediated glucose uptake in Caco-2 cells [9]. Hesperetin and homoeriodictyol are naturally produced in *Citrus* spp. [10] and *Eriodictyoln californicum* [11], respectively. However, extracting such compounds from plants poses significant challenges due to their low concentrations, making the subsequent purification and separation processes intricate, time-consuming, and costly [12].

As a promising alternative, the use of metabolic engineering and synthetic biology tools has facilitated the heterologous biosynthesis of flavonoids [13]. Different microbial hosts have been employed to produce these plant secondary metabolites, such as *Saccharomyces cerevisiae* [14], *Escherichia coli* [15], and *Streptomyces albidoflavus* [16]. Among the microorganisms utilized as microbial cell factories for the biosynthesis of flavonoids, *Streptomyces* stands out, recognized for its natural capacity to synthesize these compounds [17,18]. In order to achieve the biosynthesis of these flavonoids, the respective biosynthetic gene cluster (BGC) for each compound must be assembled using synthetic genes utilizing codon-optimized sequences for optimal expression in selected microbial hosts.

**Figure 1:**
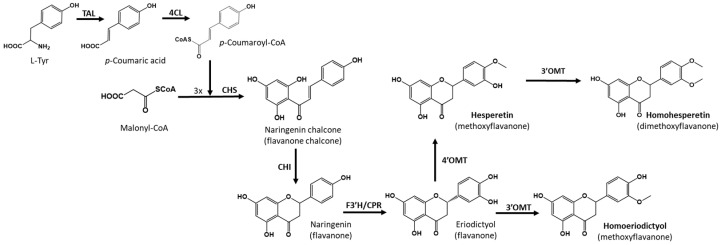
Biosynthetic pathway for the heterologous biosynthesis of hesperetin and homoeriodictyol. Tyrosine ammonia-lyase (TAL); 4-Coumaroyl-CoA ligase (4CL); Chalcone synthase (CHS); Chalcone isomerase (CHI); Flavone synthase (FNS); 4′-*O*-methyltransferase (4′OMT); 3′-*O*-methyltransferase (3′OMT); *S. albidoflavus* endogenous *O*-methyltransferase (3′OMT).

To achieve the biosynthesis of hesperetin and homoeriodictyol from the primary precursor l-tyrosine, six enzymatic steps are necessary. First, the enzyme tyrosine ammonia lyase (TAL) converts l-tyrosine to *p*-coumaric acid, which is then activated with a molecule of coenzyme A by the action of a 4-coumaroyl-CoA ligase (4CL). The next step is a Claisen condensation carried out via chalcone synthase (CHS), which condenses three molecules of malonyl-CoA with 4-coumaroyl-CoA, producing the key flavonoid intermediate, naringenin chalcone, which is finally cyclized via the action of chalcone isomerase (CHI) to yield naringenin. The flavanone naringenin is converted to eriodictyol via the action of flavanone-3′-hydroxylase (F3′H), which introduces a hydroxyl group in the 3′ position of the B ring. Finally, 4′-*O*-methyltransferase is necessary to produce hesperetin, and 3′-*O*-methyltransferase is necessary in the case of homoeriodictyol (Figure 1).

The current limitation in the heterologous biosynthesis of flavonoids lies in the low production yields associated with the limited availability of intracellular precursors and co-factors. Nowadays, metabolomics offers valuable insights into cellular metabolism, easily integrated with stable isotope tracing, flux measurements, and other “omics” tools like transcriptomics or proteomics. This integration reveals intricate metabolite dynamics and metabolic interconnections [19]. A primary tool for developing suitable microbial cell factories for the biosynthesis of specific compounds is the generation and study of transcriptomes, which allows quantitative measurements of dynamic mRNA expression levels between different conditions or states, reflecting the changes in the gene expression under particular conditions, which in turn, allows the performance of tailor-made metabolic engineering experiments [20].

So far, different studies of metabolic engineering based on transcriptomics can be found in the genus *Streptomyces.* In 2016, Kim and colleagues developed a transcriptomics-based strain optimization tool (tSOT) to predict metabolic targets to overproduce secondary metabolites in *Streptomyces coelicolor*, obtaining a 2- and a 1.8-fold increase in actinorhodin production with the overexpression of the enzyme 5-phosphate-3-epimerase and the NADP-dependent malic enzyme, respectively [21]. In another study, Yousra Ahmed and co-workers performed a transcriptomic study in *S. albidoflavus* J1074 for the identification of a butenolide regulatory system involved in the control of secondary metabolism [22]. Similarly, Wang and co-workers found that the strong promoter P*_thlM4_* was able to achieve a 30% increase in the production of the antibiotic natamycin in *Streptomyces chattanoogensis* L10 when compared with the use of the P_ermE*_ promoter [23].

In this study, we performed a comparative analysis of *S. albidoflavus* J1074 in two different conditions, feeding and not feeding, with l-tyrosine. The aim was the identification of the genes that relate to the metabolism of l-tyrosine, the first precursor in the biosynthesis of flavonoids. In previous studies, different strategies have been applied to enhance the intracellular pool of l-tyrosine for flavonoid biosynthesis, such as the removal of feedback inhibition using aromatic amino acids shown by the enzyme DAHP (3-Deoxy-D-arabinoheptulosonate 7-phosphate) synthase in the shikimate pathway, the route involved in aromatic amino acid biosynthesis [24]. Here, a transcriptomic analysis was performed to compare the gene expression in *S. albidoflavus* J1074 after the addition of l-tyrosine to the medium to find genes that negatively act in l-tyrosine biosynthesis. The analysis resulted in the identification of *hppD* as a gene that deregulates l-tyrosine biosynthesis. By knocking this out, we managed to enhance the production of the central flavonoid naringenin, and the improved *S. albidoflavus* strain was employed to biosynthesize methylated flavonoids.

## 2. Results

### 2.1. Transcriptomic Assay in S. albidoflavus When Feeding with l-tyrosine

To increase the intracellular pool of l-tyrosine towards flavonoid biosynthesis, the amino acid was supplemented to *S. albidoflavus* J1074, and a differential gene expression analysis (RNAseq) was performed versus a control *S. albidoflavus* cultivation without l-tyrosine feeding.

**Figure 2:**
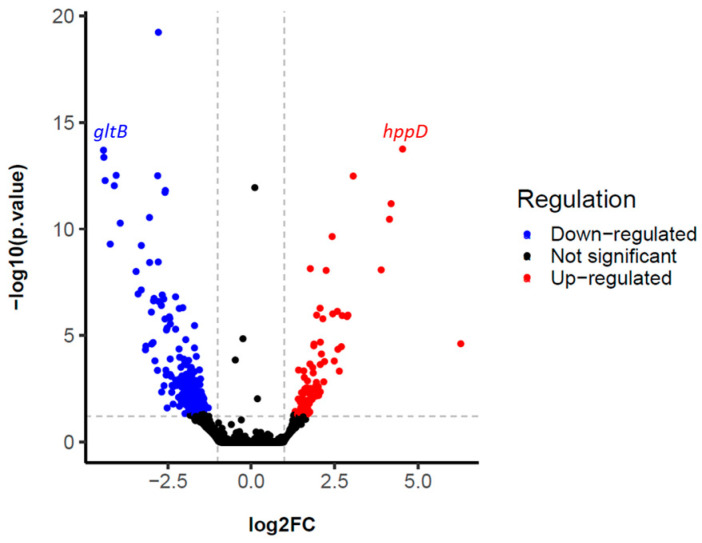
Volcano plot highlighting differentially expressed (DE) genes of the l-tyrosine-fed *S. albidoflavus* J1074 cultivation versus the control cultivation. The blue and red dots indicate the genes that are significantly down-regulated and up-regulated, respectively. The two vertical grey lines indicate the boundaries of genes with |log2FC| > 1, *p*-value < 0.05. The horizontal grey line indicates the significance threshold of 1.3, calculated using −log10 of the *p*-value.

The results showed the expression of a total of 5885 different genes. Non-specific filtering was performed to exclude those genes that were in very low abundance, obtaining a total of 5351 filtered genes. According to the established parameters (see Section 4), a total of 4115 differentially expressed (DE) genes were found, of which 285 showed deregulation after pairwise DE analysis (*p*-value < 0.05) (Figure 2 and Table S1).

**Figure 3:**
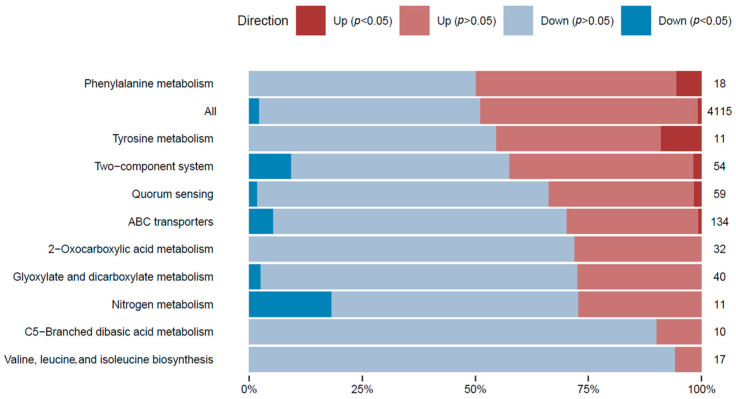
GSA of the gene expression in the l-tyrosine feeding condition. Gene sets were defined by pathway terms. The top 10 pathway terms are shown based on the significant DE genes. The number of genes with changes in the relative gene expression within each category is shown. The percentage of genes with significant DE genes (*p*-adjusted < 0.05) is shown within the bars in dark blue (repression) and dark red (overexpression). Pathway term annotations can be redundant, and the same genes could belong to different pathway terms.

In order to attain a thorough comprehension of the gene expression differences in the transcriptome analysis and to classify each expressed gene within a biological context, a gene set analysis (GSA) was executed on all differentially expressed genes. GSA is a technique used to detect groups of genes or proteins that are over-represented within a vast dataset of genetic or protein information. These identified groups may indicate potential associations with various phenotypic traits. To facilitate the analysis, genes were annotated based on their associations with metabolic pathways (Figure 3), paying special attention to those linked to l-tyrosine metabolism.

**Figure 4:**
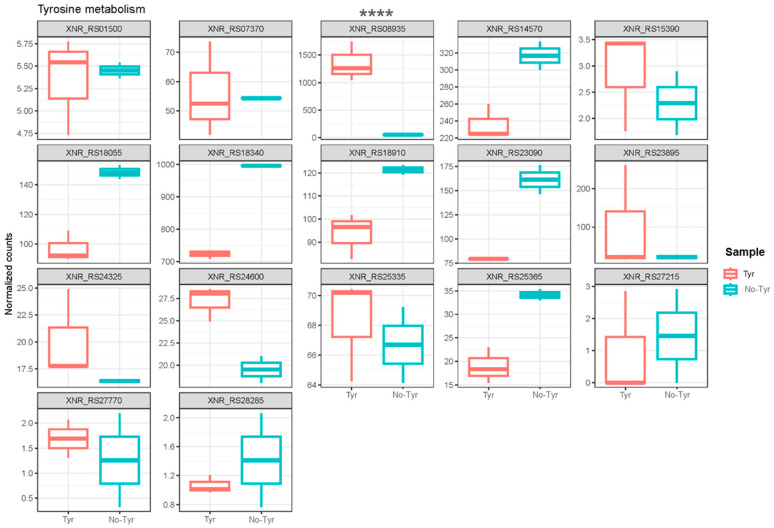
Box plots showing the normalized counts of the different genes belonging to l-tyrosine metabolism either in the l-tyrosine-fed or the non-fed cultures of *S. alibidoflavus*. XNR_RS01500: NADP-dependent succinic semialdehyde dehydrogenase; XNR_RS07370: NAD-dependent succinate-semialdehyde dehydrogenase; XNR_RS08935: 4-hydroxyphenylpyruvate dioxygenase (HppD); XNR_RS14570: histidinol-phosphate transaminase; XNR_RS15390: NAD(P)-dependent alcohol dehydrogenase; XNR_RS18055: fumarylacetoacetase; XNR_RS18340: pyridoxal phosphate-dependent aminotransferase; XNR_RS18910: succinate-semialdehyde dehydrogenase (NADP(+)); XNR_RS23090: NAD-dependent succinate-semialdehyde dehydrogenase; XNR_RS23895: histidinol-phosphate transaminase; XNR_RS24325: maleylpyruvate isomerase family mycothiol-dependent enzyme; XNR_RS24600: Rv2231c family pyridoxal phosphate-dependent protein CobC; XNR_RS25335: homogentisate 1,2-dioxygenase; XNR_RS25365: zinc-binding dehydrogenase; XNR_RS27215: FAD-dependent oxidoreductase; XNR_RS27770: alcohol dehydrogenase; XNR_RS28285: zinc-binding alcohol dehydrogenase family protein. The asterisks indicate statistically significant differences in the only gene, showing these differences between the two conditions (**** *p* <0.0001).

**Figure 5:**
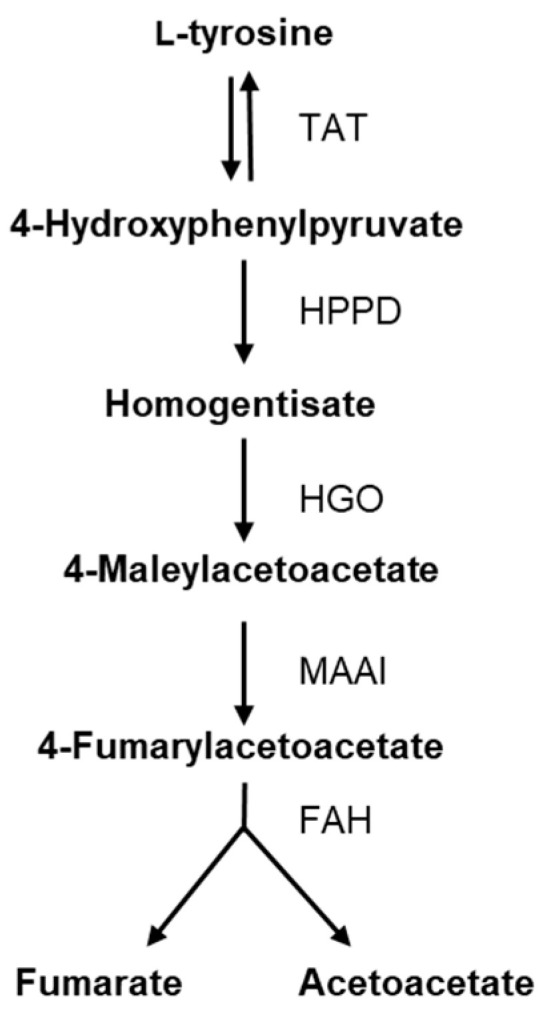
Enzymatic steps of tyrosine catabolism. TAT: tyrosine aminotransferase; HPPD: 4-hydroxyphenylpyruvate dioxygenase; HGO: homogentisate dioxygenase; MAAI: maleylacetoacetate isomerase; FAH: fumarylacetoacetate hydrolase.

To explore the genes associated with l-tyrosine metabolism, a comparison of normalized gene counts (see Section 4) within this pathway was conducted (Figure 4). Among these genes, XNR_RS08935 exhibited significant deregulation (*p*-value < 0.0001) in *S. albidoflavus* J1074 upon supplementation with l-tyrosine in the growth medium. Notably, XNR_RS08935 (encoding 4-hydroxyphenylpyruvate dioxygenase, HppD) demonstrated the highest level of expression under the feeding condition (23.2-FC) (Table S1). It is involved in the conversion of 4-hydroxyphenylpyruvate to homogentisate within the l-tyrosine catabolic pathway (Figure 5). This result suggested that in the presence of l-tyrosine in the medium, *hppD* is overexpressed to enhance the catabolism of this aromatic amino acid. Thus, the elimination of this gene from the bacterial chromosome could lead to an increase in the availability of l-tyrosine for the heterologous production of flavonoids.

### 2.2. Effect of a Knock-Out for the hppD Gene in the Biosynthesis of Naringenin

The strain of *S. albidoflavus* used in this study is a derivative version of the *S. albidoflavus* UO-FLAV-004 strain, which was previously generated for the biosynthesis of different flavonoids [25]. This strain lacks some BGCs encoding for certain secondary metabolites. In pursuit of further genome reduction in this bacterial host to mitigate competing pathways, we developed the *S. albidoflavus* UO-FLAV-005 strain, removing the BGC encoding the terpenoid isorenieratene (BGC number 20 predicted using anti-SMASH in the genome of *S. albidoflavus* J1074) from the chromosome of *S. albidoflavus* UO-FLAV-004 (Figure S1).

**Figure 6:**
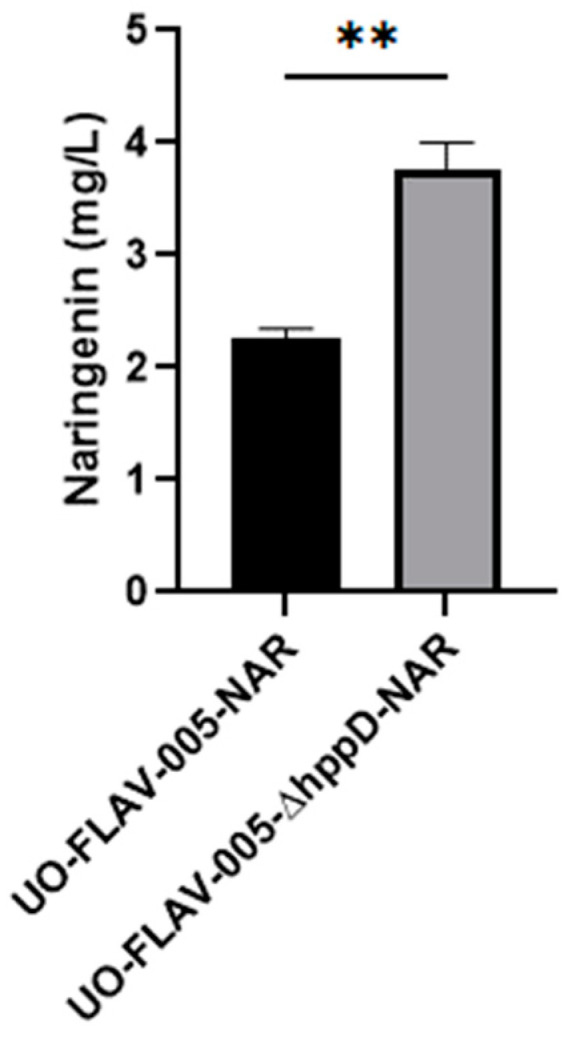
Effect of the *hppD* knock-out in the biosynthesis of naringenin in *S. albidoflavus*. The asterisks indicate statistically significant differences (** *p* < 0.005).

The *S. albidoflavus* UO-FLAV-005 strain was further engineered for the generation of a knock-out strain for the gene *hppD,* delivering *S. albidoflavus* UO-FLAV-005-∆*hppD* (Figure S2). In order to check the effect of this genetic modification on the biosynthesis of flavonoids, both *S. albidoflavus* UO-FLAV-005-∆*hppD* and *S. albidoflavus* UO-FLAV-005 strains were transformed with the plasmid pSEVAUO-M21703–NarBGC, containing the naringenin BGC, which was integrated into the ΦBT1 *attb* site of the bacterial chromosome. The resulting strains, *S. albidoflavus* UO-FLAV-005-∆*hppD*-NAR and *S. albidoflavus* UO-FLAV-005-NAR, were cultivated simultaneously in triplicates as described in Section 4. After 5 days of fermentation, the *S. albidoflavus* UO-FLAV-005-NAR strain yielded 2.25 mg/L of naringenin, while the *S. albidoflavus* UO-FLAV-005-*hppD*-NAR strain produced 3.75 mg/L, which represents a 1.66-fold increase (Figure 6 and Figure S3).

### 2.3. Heterologous Biosynthesis of Hesperetin and Homoeriodictyol

**Figure 7:**
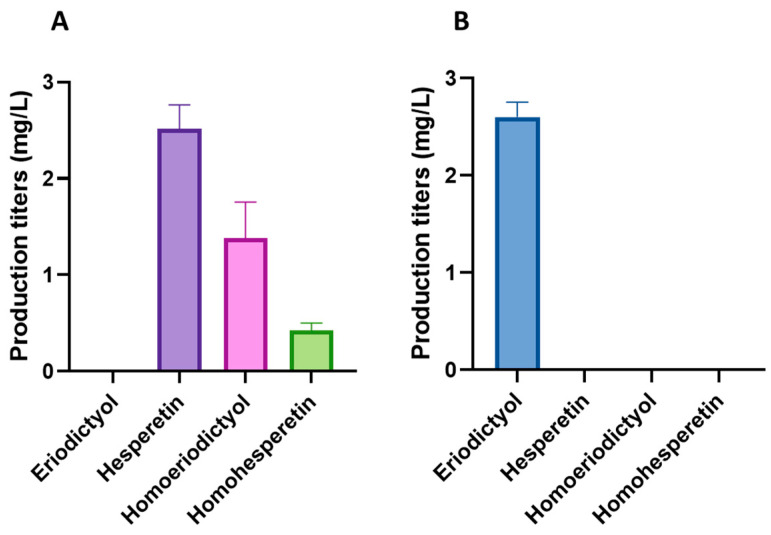
(**A**) Production titers of different flavonoids in the *S. albidoflavus* UO-FLAV-005-∆*hppD*-HES/HOM strain. (**B**) Production titers of different flavonoids in the control *S. albidoflavus* UO-FLAV-005-∆*hppD*-ERI strain.

For the biosynthesis of the methylated flavonoids hesperetin and homoeriodictyol, the improved flavonoid-producing *S. albidoflavus* UO-FLAV-005-∆*hppD*-NAR strain was transformed with the plasmid pSEVAUO-M11501-HES/HOM, which contains a chimeric F3′H-CPR enzyme [16] and a caffeoyl-coenzyme A-*O*-methyltransferase (CCoAOMT)-like enzyme encoded by the gene *At4g26220* from *Arabidopsis thaliana*, which was integrated into the ΦC31 *attb* site of the chromosome to generate the *S. albidoflavus* UO-FLAV-005-∆*hppD*-HES/HOM strain. This CCoAOMT-like enzyme is able to introduce a methyl moiety in both the 4′ and 3′ positions of eriodictyol, yielding hesperetin and homoeriodictyol, respectively. In vitro, this enzyme converts 80% of eriodictyol to hesperetin and 20% to homoeriodictyol [26]. In vivo, after 5 days of fermentation, the *S. albidoflavus* UO-FLAV-005-∆*hppD*-HES/HOM strain produced 2.52 mg/L of hesperetin and 1.38 mg/L of homoeriodictyol (Figure 7A), while the control *S. albidoflavus* UO-FLAV-005-∆*hppD*-ERI strain, harboring only the gene encoding for the chimeric F3′H-CPR moiety, produced 2.6 mg/L of eriodictyol (Figure 7B) and no hesperetin or homoeriodictyol were detected (Figure 7B). No remaining eriodictyol was detected in the extract of the *S. albidoflavus* UO-FLAV-005-∆*hppD*-HES/HOM strain, indicating the total conversion of the substrate (Figure 7A and Figure S4).

### 2.4. Identification of a di-Methylated Flavanone in the Extracts of the S. albidoflavus UO-FLAV-005-∆hppD-HES/HOM Strain

During the analysis of the biosynthesis of hesperetin and homoeriodictyol in the *S. albidoflavus* UO-FLAV-005-∆*hppD*-HES/HOM strain, a peak exhibiting a retention time other that of hesperetin and homoeriodictyol was identified, which was absent in the extract of the *S. albidoflavus* UO-FLAV-005-∆*hppD*-ERI control strain (Figure S4). The retention time and the spectrum of this compound suggested that it could be a di-methylated derivative of eriodictyol. However, although the regioselectivity of an enzyme may vary in vivo [27], the methyltransferase enzyme encoded by the gene *At4g26220* does not produce di-methylated derivatives of eriodictyol in vitro, even after long incubation times [26]. To determine whether the enzyme responsible for the production of this compound was endogenous to *S. albidoflavus*, two feeding experiments were conducted with the *S. albidoflavus* UO-FLAV-005 strain, one with hesperetin and the other with homoeriodictyol. The compound in question eluted at 35.2 min (Figure S4) and was observed when the strain was supplemented with hesperetin but not with homoeriodictyol (Figure S5). These results indicate that *S. albidoflavus* possesses an enzyme able to modify hesperetin, which is not uncommon since other flavonoid methyltransferases have been reported in other *Streptomyces* species [28,29,30,31,32]. This extra (unknown) methyl group could be located in the 7, 5, or 3′ positions of hesperetin when *S. albidoflavus* is used as a flavonoid factory. In order to identify the compound, a first trial was carried out with a pure standard of homohesperetin (also known as hesperetin 3′-methyl ether), which was analyzed using a HPLC-DAD (high-performance liquid chromatography-diode array detector). The identity of the compound was confirmed as homohesperetin via co-elution with the pure compound and by analyzing the absorption spectrum (Figure S6). The production of homohesperetin reached 0.43 mg/L in the *S. albidoflavus* UO-FLAV-005-∆*hppD*-HES/HOM (Figure 7) strain. These results indicate that *S. albidoflavus* is able to introduce a methyl moiety in the 3′ position of hesperetin (Figure 1). However, further research will be needed to identify the responsible enzyme of *S. albidoflavus* for the generation of homohesperetin.

## 3. Discussion

The objective of this study was to identify the deregulated genes that negatively act on the internal l-tyrosine pool in cases where this amino acid is present at elevated concentrations. We performed a differential gene expression analysis based on the comparison between the transcripts of *S. albidoflavus* J1074 fed with l-tyrosine and a control condition where the amino acid was not added to the medium. The first reaction in the l-tyrosine catabolic pathway is catalyzed by the enzyme tyrosine aminotransferase, which converts l-tyrosine to 4-hydroxyphenylpyruvate and vice versa. The next reaction is carried out via the HppD enzyme, which leads to the conversion of 4-hydroxyphenylpyruvate into homogentisate (Figure 5). Furthermore, HppD is involved in the L-phenylalanine metabolic pathway to convert phenylpyruvate into 2-hydroxy-phenylacetate. Thus, the deletion of *hppD* was expected to lead to the accumulation of 4-hydroxyphenylpyruvate and L-phenylalanine that could be further converted to l-tyrosine [33]. However, *S. albidoflavus* cannot perform the last interconversion.

Previously, Yang and co-workers studied the regulatory mechanism of the *hppD* gene in *Streptomyces coelicolor.* The transcription of the *hppD* gene is regulated by the transcriptional activator HpdA in response to 4-hydroxyphenylpyruvate, the molecule that interacts with the transcriptional regulator [34]. In this study, we observed that an excess of l-tyrosine in the medium promotes the overexpression of *hppD*, resulting in the degradation of this amino acid. Our hypothesis to knock out the *hppD* gene in *S. albidoflavus* is that this would potentially increase the amount of 4-hydroxyphenylpyruvate that could be converted back to l-tyrosine, avoiding its degradation towards homogentisate. Therefore, the regulatory mechanism described by Yang and collaborators would be deactivated due to the absence of *hppD,* and the intracellular pool of l-tyrosine may be kept enhanced, which would be translated into an increase in the flavonoid production titers.

Prior to the generation of a knock-out for *hppD*, we carried out the deletion of the isorenieratene BGC from the chromosome of *S. albidoflavus* UO-FLAV-004, a previously developed strain of *S. albidoflavus* with an increased malonyl-CoA pool optimized for flavonoid biosynthesis [24]. The biosynthesis of the isorenieratene terpenoid involves the utilization of acetyl-CoA to produce the dimethylallyl pyrophosphate precursor [35]. In *Streptomyces* spp., acetyl-CoA is converted to malonyl-CoA through the acetyl coenzyme A carboxylase complex [36]. Malonyl-CoA is a key intermediate in the biosynthesis of flavonoids, and its utilization in other endogenous metabolic pathways would lead to a decrease in the intracellular levels that could affect the final flavonoid production titers. The strain generated after the deletion of the isoreneriatene BGC, *S. albidoflavus* UO-FLAV-005, was used as a base to generate the knock-out strain for *hppD.*

In a previous study, Suzanne Verhoef and co-workers studied the expression changes associated with *p*-hydroxybenzoate production in an engineered strain of *Pseudomonas putida* through transcriptomics and proteomics. They observed that *hppD* was slightly up-regulated in the optimized *p*-hydroxybenzoate-producing strain compared to the non-optimized control strain when glucose or glycerol was used as the substrate. The deletion of *hppD* resulted in an increase of *p*-hydroxybenzoate of 22% and 21%, with glucose and glycerol as the substrates, respectively [37]. In this work, we present the first instance where the *hppD* gene has been knocked out as a strategy to increase the biosynthesis of flavonoids in a microbial factory, showing a significant increase of 66% in naringenin biosynthesis, supporting this strategy as an effective metabolic engineering approach to increase the biosynthesis of l-tyrosine-derived compounds.

As the *S. albidoflavus* UO-FLAV-005-∆*hppD*-NAR strain was a significantly better flavonoid producer than the parental *S. albidoflavus* UO-FLAV-005 strain, this strain was employed to produce hesperetin and homoeriodictyol, two methylated derivatives of eriodictyol, with a methyl moiety in the 4′ and 3′ positions, respectively. Previously, hesperetin was produced in an engineered *E. coli* consortium fed with naringenin [38]. This *E. coli* consortium consisted of a first recombinant host harboring the 3′-hydroxylase (F3′H) flavonoid from *Gentiana triflora* and cytochrome P_450_ reductase (CPR) from *Arabidopsis thaliana* and a second strain containing the 4′-*O*-methyltransferase flavonoid from *Mentha* × *piperita*. In optimal conditions, the hesperetin titers reached 37.1 mg/L after feeding with 50 mg/L of naringenin [38]. On the other hand, homoeriodictyol was produced in recombinant *E. coli* by adding *p*-coumaric acid to the culture medium, reaching 52 mg/L from 164 mg/L of *p*-coumaric acid [39]. Additionally, hesperetin and homoeriodictyol were recently produced in *E. coli* from caffeic acid, reaching titers of 14.6 mg/L and 3.8 mg/L, respectively [40]. In this study, unlike the previous reports, we present for the first time the complete de novo biosynthesis of hesperetin and homoeriodictyol in a heterologous host.

In summary, it has been proved that *S. albidoflavus* is a suitable host for the heterologous biosynthesis of methylated flavonoids. Additionally, these results highlight the potential of this bacterium for the production of methylated flavonoids using endogenous enzymes. In this case, an unexpected and unwanted flavonoid derivative (homohesperetin) was produced, interfering with the biosynthesis of the target compounds (hesperetin and homoeriodictyol) and reducing their titers. Therefore, a good strategy to increase flavonoid production levels is the identification and deletion of the native genes that are naturally present in the genome of *S. albidoflavus* and can modify the final target compounds. Moreover, the identification of these genes would allow for their characterization and use for the biosynthesis of modified natural products. This potential of *S. albidoflavus* to synthesize flavonoid derivatives like homohesperetin using its intrinsic cellular mechanisms represents an interesting characteristic that may facilitate the future generation of novel flavonoids through the identification and heterologous expression of native genes.

## 4. Materials and Methods

### 4.1. Reagents and Biochemicals

All of the solvents used for the solid phase extraction and HPLC-DAD analysis were LC-MS (liquid chromatography-mass spectroscopy) grade from Sigma-Aldrich (Madrid, Spain) or VWR Chemicals (Barcelona, Spain). Naringenin, eriodictyol, hesperetin, and homoeriodictyol were provided by Extrasynthese (Genay, France), while l-tyrosine (dissolved in distilled water and filtered using 0.2 μm nylon filters, VWR, Barcelona, Spain) was provided by Sigma-Aldrich (Madrid, Spain). PCRBIO Ladder II (Sursee, Switzerland) was used in the agarose gels for the PCR experiments.

### 4.2. Genes and Enzymes

The restriction enzymes and T4 DNA ligase were purchased from Thermo Fisher Scientific (Madrid, Spain). Herculase II Fusion DNA polymerase was purchased from Agilent Technologies (Madrid, Spain), Terra PCR Direct polymerase was purchased from Takara (Saint-Germain-en-Laye, France), and the NEBuilder^®^ HiFi DNA Assembly Master Mix was purchased from New England BioLabs (Ipswich, MA, USA). A synthetic gene for the *Arabidopsis thaliana* caffeoyl CoA methyltransferase (At4g26220) enzyme was synthesized by EXPLORA BIOTECH (Venezia, Italy) after codon optimization (GenBank reference OR820609). All of the primers used are listed in Table S2.

### 4.3. Bacterial Strains and Culture Conditions

All of the strains used in this study are listed in Table 1. *Escherichia coli* TOP10 (Invitrogen, Waltham, MA, USA) was used for routine subcloning. *E. coli* ET12567/pUZ8002 [41] was used for conjugation. All of the *S. albidoflavus* strains presented in this work were generated via bacterial conjugation and were delivered via the engineering of the *S. albidoflavus* J1074 parent strain [42]. All feeding experiments for hesperetin and homoeriodictyol were performed with these strains. The new strains were confirmed via antibiotic resistance tests and, in the case of chromosomal deletions, via a polymerase chain reaction (PCR). The production strains were further analyzed for the biosynthesis of the desired compounds. For the conjugation of *S. albidoflavus*, the samples were grown at 30 °C in MA medium [43]. The *S. albidoflavus* strains were cultured for sporulation in Bennet medium [44] and supplemented with the corresponding antibiotics when necessary (thiostrepton 50 µg/mL or apramycin 50 µg/mL, Cayman Chemical, MI, USA). Production cultivations were initiated with 10^6^ spores/mL in 25 mL of NL333 medium (in triplicates) and incubated for 120 h at 30 °C and 250 rpm.

For the transcriptomic assay, *S. albidoflavus* J1074 was cultivated in NL333 medium [44] in triplicates for each condition (with and without supplemented l-tyrosine). In the feeding experiments, l-tyrosine was added to the flasks 6 h (before the onset of flavonoid biosynthesis in this strain [46]), after the inoculation of the culture, reaching a final concentration of 1.5 mM.

The *E. coli* strains were grown in tryptic soy broth (TSB, VWR, Barcelona, Spain) or on TSB agar plates supplemented with the corresponding antibiotic (ampicillin 100 µg/mL, Sigma Aldrich, Madrid, Spain; apramycin 100 µg/mL, Thermo Fisher Scientific, Waltham, MA, USA; and X-gal (AppliChem, Darmstadt, Germany)) when blue–white selection was needed.

### 4.4. Engineering of the S. albidoflavus Strains

#### 4.4.1. Construction of the Flavonoid-Producing Strains

The strain used to knock out the *hppD* gene was *S. albidoflavus* UO-FLAV-005, a mutant of *S. albidoflavus* UO-FLAV-004 [25] that lacks the endogenous BGC of the terpenoid isorenieratene, which was generated using the pSEVAUO-C41012-BGC20 plasmid. The *S. albidoflavus* UO-FLAV-005 strain was transformed with the pSEVAUO-C41012-∆*hppD* plasmid for the *hppD* knock-out experiment, yielding the *S. albidoflavus* UO-FLAV-005-∆*hppD* strain. To reconstitute the naringenin BGC, the pSEVAUO-M21703–NarBGC plasmid [46] was integrated into the ΦBT1 *attb* site of the chromosome, either in *S. albidoflavus* UO-FLAV-005 or *S. albidoflavus* UO-FLAV-005-∆*hppD*, producing *S. albidoflavus* UO-FLAV-005-NAR and *S. albidoflavus* UO-FLAV-005-∆*hppD*-NAR, respectively.

This last strain was further engineered to produce the methylated flavonoid derivatives hesperetin and homoeriodictyol. The extra enzymes needed to achieve the biosynthesis of only the two compounds were a F3′H-CPR chimera and a CCoAOMT-like methyltransferase enzyme. The respective genes were integrated into the φC31 *attb* site of *S. albidoflavus* UO-FLAV-005-∆*hppD*-NAR. The new strain generated, *S. albidoflavus* UO-FLAV-005-∆*hppD*-HES/HOM, drives the biosynthesis of hesperetin and homoeriodictyol.

An eriodictyol-producing strain was generated as a control for the biosynthesis of these two methylated flavonoids with the integration of the *F3′H-CPR* coding gene into the φC31 *attb* site of *S. albidoflavus* UO-FLAV-005-∆*hppD*-NAR, yielding *S. albidoflavus* UO-FLAV-005-∆*hppD*-ERI.

#### 4.4.2. The Deletion of BGC Number 20 and Generation of the *hppD* Knock-Out Mutant Strain

For the deletion of the isorenieratene-encoding BGC, a protospacer of 20 base pairs (bp) comprising the chromosomal positions of 6,412,606–6,412,625 (the reference genome of *Streptomyces albidoflavus* J1074 (accession number: NC_020990)) was cloned into the CRISPR-based plasmid pSEVAUO-C41012 [16] via a golden gate reaction, generating pSEVAUO-C41012-Spacer-BGC20.

In the same manner, for the generation of the *hppD* knock-out strain, a protospacer of 20 bp comprising the chromosomal positions of 2,123,835–2,123,854 was cloned in the pSEVAUO-C41012 plasmid, producing the pSEVAUO-C41012-Spacer-∆*hppD* plasmid.

These two plasmids contain the sgRNA and the Cas9 protein to direct a precise double-strand break over the desired chromosomal regions. To obtain a final version of each of them with the necessary elements to repair the DNA damage generated from the Cas9 protein, two homologous arms flanking the BGC of isorenieratene (in the case of BGC20) and two homologous arms flanking the core of the *hppD* gene (one of the regions contains part of the beginning of this gene and the other part of the end of this gene) were amplified from the *S. albidoflavus* genome using HerculaseII Fusion DNA polymerase and cloned into the pSEVA88c1 intermediate plasmid [16] via Gibson assembly, producing the pSEVA88c1-BGC20 and pSEVA88c1-∆*hppD* plasmids, respectively.

The homologous region A of pSEVA88c1-BGC20 comprised the chromosomal regions from 6,402,471 to 6,404,562, while the homologous region B covered the region between the chromosomal regions from 6,422,621 to 6,424,810.

In the case of pSEVA88c1-∆*hppD*, the homologous region A comprised the chromosomal coordinates of 2,119,953–2,123,029, and the homologous region B comprised the chromosomal regions of 2,123,962–2,128,100.

Finally, for the generation of the final plasmids for genome editing, the corresponding homologous arms of the pSEVA88c1-BGC20 and pSEVA88c1-∆*hppD* intermediate plasmids were cloned into the pSEVAUO-C41012-Spacer-BGC20 and pSEVAUO-C41012-Spacer-∆*hppD* plasmids, respectively, using the *PacI* and *SpeI* restriction enzymes and T4 DNA ligase, leading to the final pSEVAUO-C41012-BGC20 and pSEVAUO-C41012-∆*hppD* plasmids, respectively.

#### 4.4.3. Construction of the Plasmids for the Biosynthesis of Hesperetin and Homoeriodictyol

The *At4g26220* gene, prepared for MoClo (modular cloning) assembly [48], was cloned by EXPLORA BIOTECH (Venezia, Italy) into the pSEVA181 plasmid, creating the MoClo level 0 pSEVA181-At4g26220 plasmid. The pSEVAUO-M21102-At4g26220 plasmid was assembled in a level 1 reaction from the pSEVA181SP25, pSEVA181RiboJ-RBS, pIDTSMARTttsbib [16], pSEVA181-At4g26220 (this study) level 0 plasmids and the pSEVAUO-M21102 level 1 receptor [16].

The pSEVAUO-M21102-At4g26220 plasmid was cloned in a level 2 MoClo reaction with the pSEVAUO-M21206F3′H-CPR level 1 plasmid [16], which contains the chimeric *F3*′*H-CPR* geneand the pSEVAUO-M11501 level 2 MoClo receptor [16], producing pSEVAUO-M11501-HES/HOM.

### 4.5. Transcriptomic Analysis

#### 4.5.1. Total RNA Extraction

The total RNA extraction, library preparation, and sequencing of inoculum of 10^6^ spores/mL was performed in flasks with NL333 medium and were incubated for 18 h at 30 °C and 250 rpm. After incubation, 1 mL of the homogenized culture was transferred into a 2 mL pre-chilled tube containing glass beads with a diameter of 500–700 μm (Thermo Fisher Scientific, Madrid, Spain) for mechanical lysis. To maintain the RNA integrity, one volume of RNA was added later (Sigma Aldrich, Madrid, Spain) per two volumes of culture. The tubes were centrifuged at 12,000 rpm in a pre-chilled centrifuge, and the supernatant was discarded. From this point, the samples were processed using the GenElute™ Total RNA Purification Kit (Sigma Aldrich, Madrid, Spain). The RNA concentration and RNA integrity number (RIN) were measured using a Bioanalyzer 2100 (Agilent Technologies, Madrid, Spain).

The stranded library preparation was carried out with the RNA samples using the Illumina Stranded Total RNA Prep with Ribo-Zero Plus (Illumina, 20040529, San Diego, CA, USA). This included the elimination of the ribosomal RNA, fragmentation, cDNA synthesis, adapter ligation, amplification, and the purification of the strand-specific libraries. Library indexing was carried out using the kit IDT for Illumina RNA UD Indexes (Illumina, 20040553, San Diego, CA, USA). TapeStation (Agilent, Madrid, Spain) was used to determine the library concentration and profile, and their quantification was carried out using Qubit (Thermo Fisher Scientific, Madrid, Spain). The generated libraries were normalized and combined in equimolecular concentrations for the optimal generation of DNA clusters. Paired-end sequencing (2 × 100bp) of the previously enriched, indexed, and multiplexed libraries was performed on the NovaSeq 6000 high-throughput platform (Illumina Inc., San Diego, CA, USA), with a minimum of 20 M PE reads (10 + 10) per sample and a read quality of 85% > Q30.

#### 4.5.2. Transcriptomic Data Analysis

The differential gene expression analysis was carried out on data generated on Illumina Novaseq using the reference genome of *Streptomyces albidoflavus* J1074 (accession number: NC_020990). The exploratory analysis was carried out using the edgeR algorithm for R [49]. Specifically, a pairwise differential expression (DE) analysis was performed after a trimmed mean of M values (TMM) normalization, a method that adjusts the library sizes under the assumption that the majority of genes do not exhibit differential expression. With the aim of maximizing the number of DE genes between conditions, the cut point of the measure of effect was established at *log2FC* > 1, correcting for multiple comparisons of the significance level using the false discovery rate (FDR) [50] at *p* < 0.05 (adjusted *p*-value). The GSA was carried out using the piano package for R [51], and the gene ontology (GO) terms and pathway terms were obtained from KEGG [52]. The script used for the transcriptomic analysis was deposited in the ZENODO database with a DOI number of 10.5281/zenodo.10877736 (URL (accessed on 4 April 2024) https://zenodo.org/records/10877736).

### 4.6. Flavonoid Extraction and HPLC-DAD Analysis

*S. albidoflavus* strains were incubated at 30 °C and 250 rpm. The flavonoids were extracted as described before [53]. HPLC-DAD analyses were carried out after resuspending the dry extracts in 100 µL of DMSO/MeOH 1:1 (*v*/*v*). The separation was performed using HPLC (1260 Infinity, Agilent Technologies) equipment and a Pursuit XRs C18 (50 × 4.0 mm, 5 μm, Agilent Technologies, Madrid, Spain) column, with the liquid phases being MeCN 0.1% formic acid (FA) and water 0.1% FA. The flow rate was set to 1 mL/min and the column temperature was 30 °C. The separation program consisted of a 10% MeCN step (min 0 to min 5.44, isocratic run) and then a linear gradient from 10% to 35% of MeCN (min 5.44 to min 21.77). After that, a linear gradient from 35% to 100% MeCN (min 27.21 to 43.54) and an isocratic step (until min 55) were applied. Finally, a gradient from 100% to 10% MeCN (min 55 to min 56) was carried out and maintained at 10% MeCN until min 61. Compass DataAnalysis 4.3 (Bruker) was used for the spectra analyses on the chromatograms extracted at a wavelength of 280 nm. Commercial standards were used for the flavonoid identification and quantification (using a calibration curve). Three biological replicates were used in all the experiments. Data are shown in mg/L.

### 4.7. Statistical Analysis

A two-way ANOVA (analysis of variance Sidak’s multiple comparisons test) was used for testing the differences in the biosynthesis of naringenin among the *S. albidoflavus* UO-FLAV-005-NAR and *S. albidoflavus* UO-FLAV-005-NAR-∆*hppD* strains. The graphical representation of the generated data was carried out using GraphPad Prism software (version 9.0.2, GraphPad Software, San Diego, CA, USA), considering a *p*-value < 0.05 as statistically significant (* *p* < 0.05; ** *p* < 0.005; *** *p* < 0.0005; **** *p* < 0.0001).

## 5. Conclusions

Transcriptomic studies are helpful for tailor-made metabolic engineering, guiding in the targeting of genes for their deletion or overexpression. The generation of a knock-out strain for the *hppD* gene is an optimal strategy to increase the biosynthesis of flavonoids, most probably due to an enhancement in the l-tyrosine intracellular pools. Moreover, the successful de novo biosynthesis of hesperetin and homoeriodictyol was demonstrated in *S. albidoflavus*, rendering this microorganism a good candidate for the production of methylated flavonoids. In addition, the metabolic potential of *S. albidoflavus* for the generation of methylated flavonoid derivatives like homohesperetin using intracellular machinery was demonstrated. However, this potential for the generation of flavonoid diversity may be complex due to the generation of undesirable shunt products that reduce the final titers of the targeted compounds.

## Figures and Tables

**Table 1:** Bacterial plasmids and strains used in this study.

Plasmids	Description	Source
pSEVA88c1	Replicative shuttle vector	[45]
pSEVA88c1-BGC20	pSEVA88c1 harboring homologous arms for BGC20 deletion	This study
pSEVA88c1-∆*hppD*	pSEVA88c1 harboring homologous arms for *hppD* deletion	This study
pSEVAUO-C41012	Replicative shuttle vector harboring the nuclease cas9	[16]
pSEVAUO-C41012-Spacer-BGC20	pSEVAUO-C41012 harboring the protospacer for BGC20 deletion	This study
pSEVAUO-C41012-Spacer-∆*hppD*	pSEVAUO-C41012 harboring the protospacer for *hppD* deletion	This study
pSEVAUO-C41012-BGC20	pSEVAUO-C41012-Spacer-BGC20 harboring homologous arms for BGC20 deletion	This study
pSEVAUO-C41012-∆*hppD*	pSEVAUO-C41012-Spacer-∆*hppD* harboring homologous arms for *hppD* deletion	This study
pSEVAUO-M21703–NarBGC	Level 2 MoClo plasmid harboring *TAL*, *4CL*, *CHS,* and *CHI*	[46]
pSEVA181-At4g26220	Source of *At4g26220* (level 0 MoClo)	This study
pSEVA181SP25	Source of *SP25* (level 0 MoClo)	[16]
pSEVA181RiboJ-RBS	Source of *RiboJ-RBS* (level 0 MoClo)	[16]
pIDTSMARTttsbib	Source of *ttsbib* (level 0 MoClo)	[16]
pSEVAUO-M21206F3′H-CPR	Level 1 MoClo harboring *F3*′*H-CPR* gene	[16]
pSEVAUO-M21102	Level 1 MoClo receptor	[16]
pSEVAUO-M21102-At4g26220	Level 1 MoClo harboring *At4g26220*	This study
pSEVAUO-M11501	Level 2 MoClo receptor	[16]
pSEVAUO-M11501pSEVAUO-M11501-HES/HOM	Level 2 MoClo plasmid harboring *F3*′*H-CPR* and *At4g26220* genes	This study
**Strains**		
*E. coli* TOP10	Strain used for routine subcloning	Invitrogen
*E. coli* ET12567/pUZ8002	Strain used for conjugation	[41]
*Streptomyces albidoflavus* J1074	*S. albidoflavus* mutant of the *S. albidoflavus* G strain that lacks an active *Sal*I restriction-modification system	[47]
*S. albidoflavus* UO-FLAV-004	*S. albidoflavus* strain used for BGC20 deletion	[25]
*S. albidoflavus* UO-FLAV-005	*S. albidoflavus* UO-FLAV-004 lacking BGC20	This study
*S. albidoflavus* UO-FLAV-005-NAR	*S. albidoflavus* UO-FLAV-005 harboring *TAL*, *4CL*, *CHS,* and *CHI*	This study
*S. albidoflavus* UO-FLAV-005-∆*hppD*-NAR	*S. albidoflavus* UO-FLAV-005-∆*hppD* harboring *TAL*, *4CL*, *CHS,* and *CHI*	This study
*S. albidoflavus* UO-FLAV-005-∆*hppD*-ERI	*S. albidoflavus* UO-FLAV-005-∆*hppD* harboring *TAL*, *4CL*, *CHS*, *CHI,* and *F3*′*H-CPR* genes	This study
*S. albidoflavus* UO-FLAV-005-∆*hppD*-HES/HOM	*S. albidoflavus* UO-FLAV-005-∆*hppD* harboring *TAL*, *4CL*, *CHS*, *CHI*, *F3*′*H-CPR,* and *At4g26220* genes	This study

## Data Availability

The data and materials can be obtained from the research group upon request. Sequence accession data have been included in Section 4.

## Supplementary Materials

The following supporting information can be downloaded at: https://www.mdpi.com/article/10.3390/ijms25074053/s1.
