# Supplementary material for: Biosynthesis of Hesperetin, Homoeriodictyol, and Homohesperetin in a Transcriptomics-Driven Engineered Strain of Streptomyces albidoflavus
Source: Int J Mol Sci. 2024 Apr 5;25(7):4053. doi: 10.3390/ijms25074053 (PMC11012174; doi:10.3390/ijms25074053)
Supplement: Supplementary file 1 [file ijms-25-04053-s001.zip › ijms-2929562-supplementary.pdf]

# Biosynthesis of Hesperetin, Homoeriodictyol and Homohesperetin in a Transcriptomics-driven Engineered Strain of *Streptomyces albidoflavus*

**Álvaro Pérez-Valero[1,2,3], Juan Serna-Diestro[1,2,3], Albert E. Tafur Rangel[4,5], Simona Barbuto Ferraiuolo[6], Chiara Schiraldi[6], Eduard J. Kerkhoven[4,5,7], Claudio J. Villar[1,2,3], Felipe Lombó[1,2,3] ***

1 Research Group BIONUC (Biotechnology of Nutraceuticals and Bioactive Compounds), Departamento de Bi-ología Funcional, Área de Microbiología, Universidad de Oviedo, Oviedo, Principality of Asturias, Spain
2 IUOPA (Instituto Universitario de Oncología del Principado de Asturias), Principality of Asturias, Spain.
3 ISPA (Instituto de Investigación Sanitaria del Principado de Asturias), Principality of Asturias, Spain.
4 Department of Biology and Biological Engineering, Chalmers University of Technology, SE-412 96 Gothenburg, Sweden.
5 Novo Nordisk Foundation Center for Biosustainability, Technical University of Denmark, DK-2800 Kgs. Lyngby, Denmark.
6 Department of Experimental Medicine, Section of Biotechnology and Molecular Biology, University of Campania "Luigi Vanvitelli", Via De Crecchio 7, 80138, Naples, Italy.
7 SciLifeLab, Chalmers University of Technology, SE-412 96 Gothenburg, Sweden.
* Correspondence: lombofelipe@uniovi.es

Supplementary Materials:

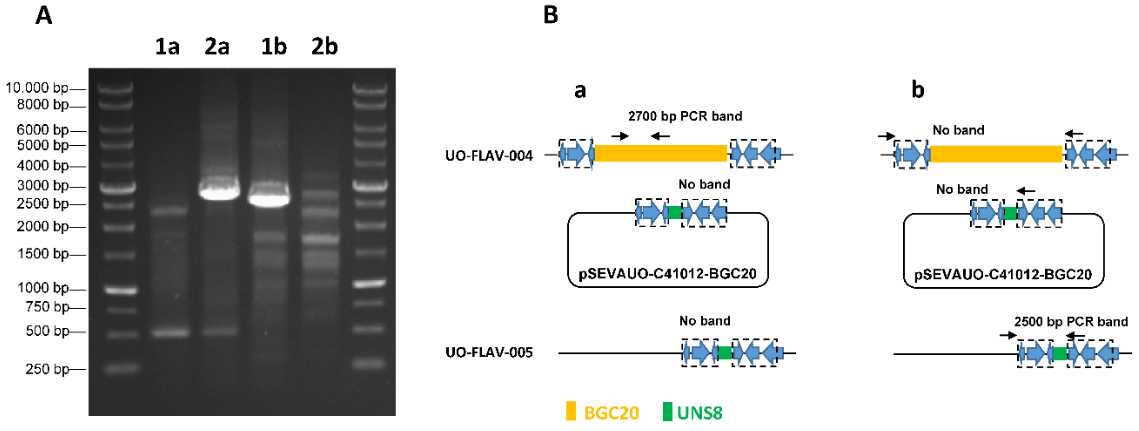


Figure S1. Generation of the *S. albidoflavus* UO-FLAV-005 strain. A) Agarose gel for PCR verification of the BGC20 deletion event, using the primers "JSD UP-checkingOK-BGC20" and "JSD RP-checkingWT-BGC20" on the mutant strain *S. albidoflavus* UO-FLAV-005 (lane 1a) and in the parental strain *S. albidoflavus* UO-FLAV-004 (lane 2a), and also using the primers "JSD UP-checkingOK-BGC20" and "JSD RP-checking-BGC20" on the mutant strain *S. albidoflavus* UO-FLAV-005 (lane 1b) and on the parental strain *S. albidoflavus* UO-FLAV-004 (lane 2b). B) Graphical representation of the expected PCR amplifications shown in the agarose gel picture: (a) expected PCR results on the parental (*S. albidoflavus* UO-FLAV-004) and mutant (*S. albidoflavus* UO-FLAV-005) strains using

the primers "JSD UP-checkingOK-BGC20" and "JSD RP-checkingWT-BGC20" (2700 bp); (b) expected PCR results with primers "JSD UP-checkingOK-BGC20" and "JSD RP-checking-BGC20" (2500 bp). The black arrows indicate the primers and are shown where annealing is possible for one or both of them. UNS8: non-coding DNA region of 40 bp in the pSEVAUO-C41012-BGC20 between the homologous arms. PCR conditions using Terra™ PCR Direct Polymerase: 1 cycle of 98 ºC (2 min), and 35 cycles of 98 ºC (10 sec) + 68 ºC (3 min).

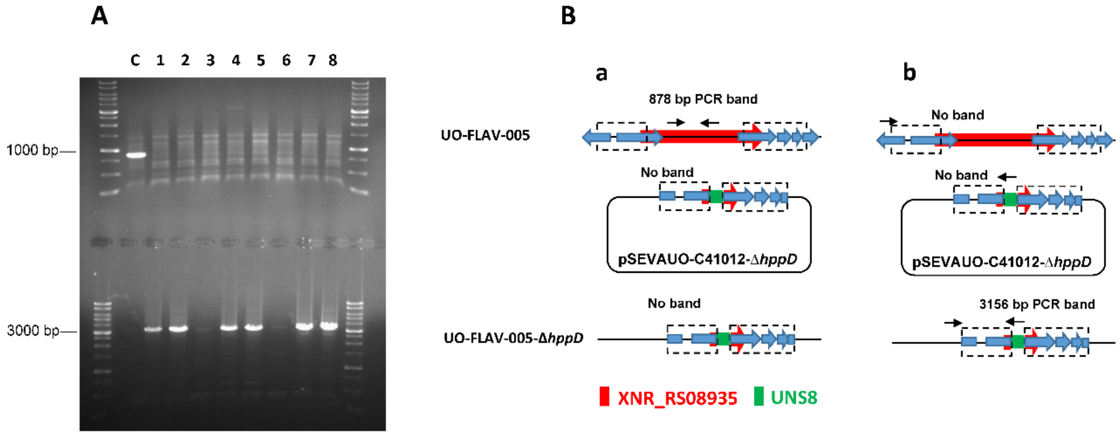


Figure S2: Generation of the *S. albidoflavus* UO-FLAV-005-Δ*hppD* strain. A) Agarose gel for PCR verification of the hppD knock-out event. Lanes on top are PCR results using the primers "Deletion hppD fw" and "Deletion hppD rev" on the mutant strain *S. albidoflavus* UO-FLAV-005-Δ*hppD* (lanes 1-8) and in the parental strain *S. albidoflavus* UO-FLAV-005 (lane C). Lanes below are PCR results using the primers "Recombination hppD FW" and "UNS8 REV" on the mutant strain *S. albidoflavus* UO-FLAV-005-Δ*hppD* (lanes 1-8) and on the parental strain *S. albidoflavus* UO-FLAV-005 (lane C). All the clones checked are correct. B) Graphical representation of the expected PCR amplifications shown in the agarose gel picture: (a) expected PCR results on the parental (*S. albidoflavus* UO-FLAV-005) and mutant (*S. albidoflavus* UO-FLAV-005-Δ*hppD*) strains using the primers "Deletion hppD fw" and "Deletion hppD rev" (878 bp); (b) expected PCR results with primers "Recombination hppD FW" and "UNS8 REV" (3156 bp). The black arrows indicate the primers and are shown where annealing is possible for one or both of them. UNS8: non-coding DNA region of 40 bp in the pSEVAUO-C41012-Δ*hpp*D between the homologous arms. PCR conditions using Terra™ PCR Direct Polymerase: 1 cycle of 98 ºC (2 min), and 35 cycles of 98 ºC (10 sec) + 68 ºC (3.5 min).

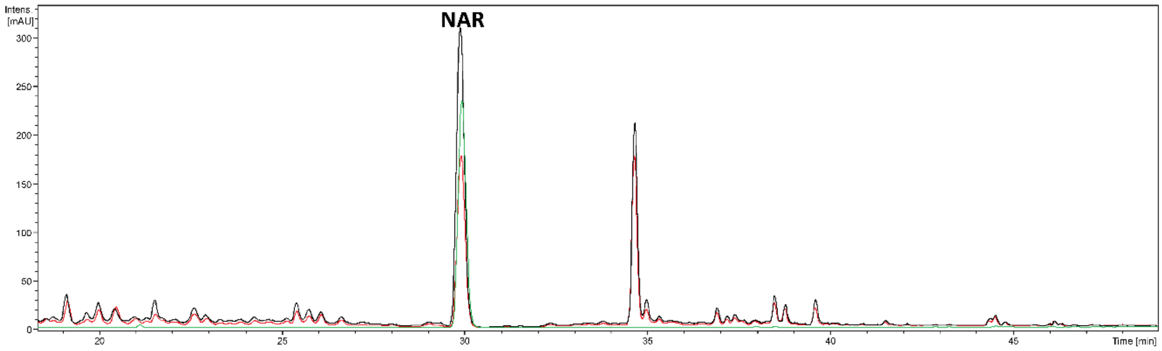


Figure S3: HPLC-DAD chromatograms showing the biosynthesis of naringenin in the strains *S. albidoflavus* UO-FLAV-005-NAR (red) and *S. albidoflavus* UO-FLAV-005-Δ*hppD*-NAR (black). Naringenin (NAR) pure standard in green.

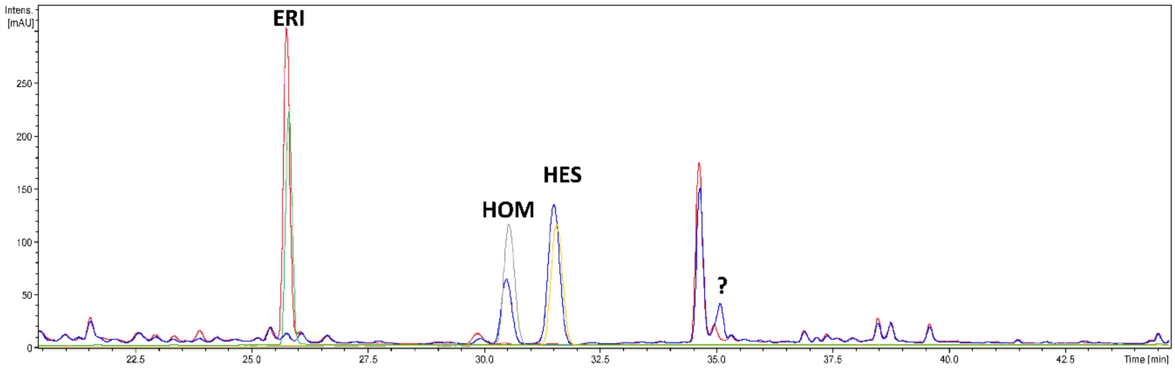


Figure S4: HPLC-DAD chromatograms of *S. albidoflavus* UO-FLAV-005-Δ*hppD*-ERI (red) and *S. albidoflavus* UO-FLAV-005-Δ*hppD*-HES/HOM (blue). Eriodictyol (ERI) pure standard in green; Homoeriodictyol (HOM) pure standard in gray; Hesperetin (HES) pure standard in orange; Tentative dimethylated eriodictyol (?).

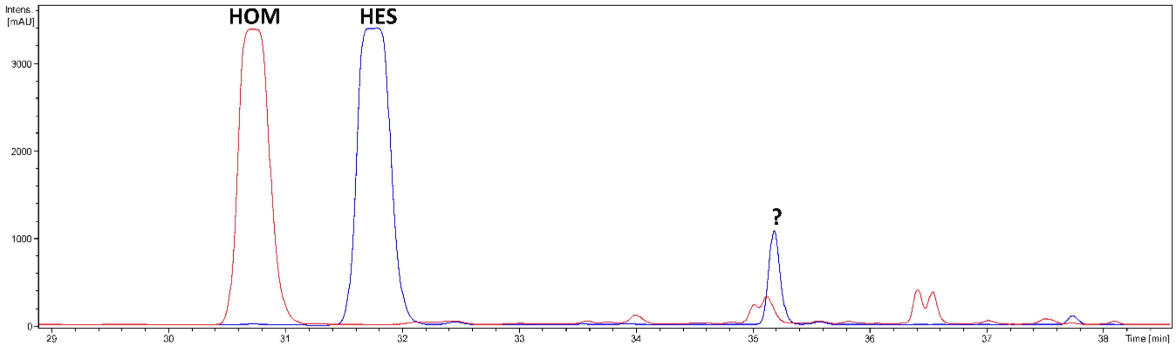


Figure S5: HPLC-DAD chromatograms of *S. albidoflavus* UO-FLAV-005 fed with homoeriodictyol (red) and hesperetin (blue). Homoeriodictyol (HOM); Hesperetin (HES); Tentative dimethyl eriodictyol (?).

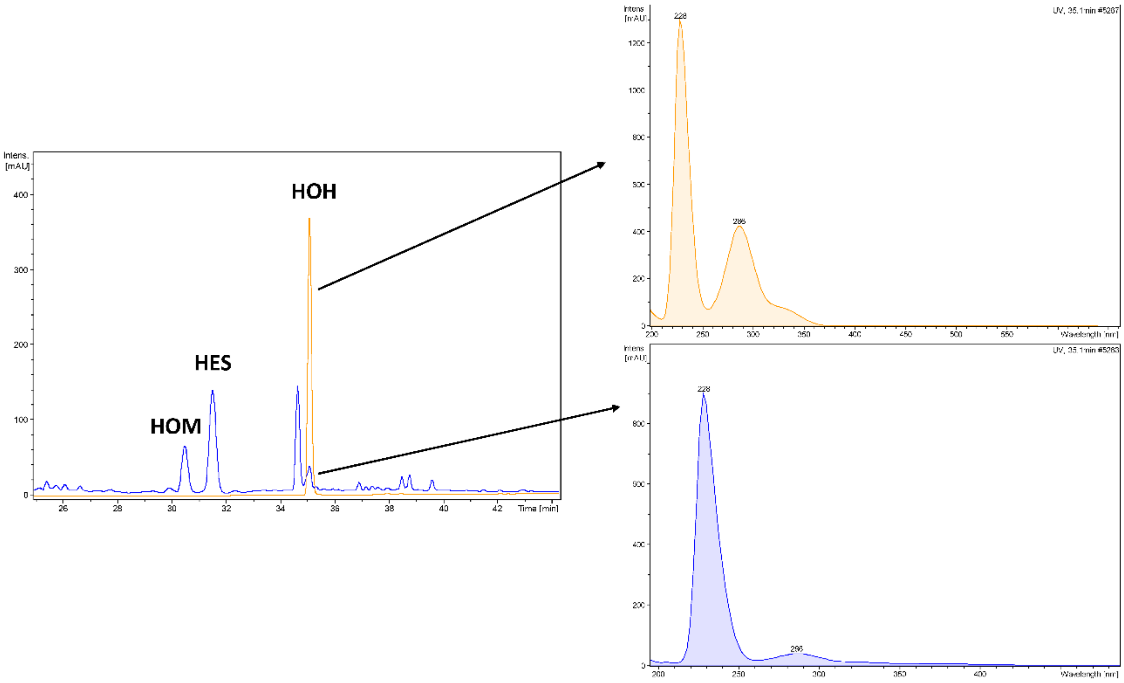


Figure S6: HPLC-DAD chromatograms *S. albidoflavus* UO-FLAV-005-Δ*hppD*-HES/HOM (blue) and a pure standard of Homohesperetin (orange). Black arrows indicate the absorption spectra of the tentative dimethyl eriodictyol (blue) and Homohesperetin (orange). Homoeriodictyol (HOM); Hesperetin (HES); Homohesperetin (HOH).

Table S1: List of differentially expressed genes. Abbreviations: WT.Tx: wild-type cultures of *S. albidoflavus* with added L-Tyr, WT-x: control cultures of the wild-type strain.

| FeatureID | Regulation | baseMean | log2FC | SE | p.value | p.adjusted | FC | Product | WT.T1 | WT.T2 | WT.T3 | WT.1 | WT.2 | WT.3 |
|---|---|---|---|---|---|---|---|---|---|---|---|---|---|---|
| [illegible] | [illegible] | [illegible] | [illegible] | [illegible] | [illegible] | [illegible] | [illegible] | [illegible] | [illegible] | [illegible] | [illegible] | [illegible] | [illegible] | [illegible] |

Table S2: Primers used in this study.

| Function | Name | Sequence 5′- 3′ |
|---|---|---|
| Homology region A BGC20 | JSD-BGC20-up1 | CAAGACGCTGGCTCTGACATTTCCGCTACTGAACTACTCGCTGGCGGACAGCAGGGGC |
| | JSD-BGC20-rp2 | CCAGGTGGTTGATGGGTTGATTGCTTTGGTTGAGACGAGGATCAGCGCGTTCACCGGC |
| Homology region B BGC20 | JSD-BGC20-up3 | CCTCGTCTCAACCAAAGCAATCAACCCATCAACCACCTGGTGCCGTGTTCCAGGCTCGTGCC |
| | JSD-BGC20-rp4 | TATGTGACCGTAGAGTATTCTTAGGTGGCAGCGAACGAGACGGTCGAGGGGAGCGTCGC |
| Recombination checking BGC20 | JSD RP-checking-BGC20 | TTGGAGTAGAGCCGTTCGAT |
| | JSD UP-checkingOK-BGC20 | GGTCACCGGTGTCGATAGTC |
| Deletion checking BGC20 | JSD UP-checkingOKBGC20 | GGTCACCGGTGTCGATAGTC |
| | JSD RP-checkingWTBGC20 | GTCTACACCACGGCGTTCAT |
| Protospacer BGC20 | JSD-BGC20-PS-up | ACGCCGCGCACCAGGTTGCGGGGG |
| | JSD-BGC20-PS-rp | AAACCCCCCGCAACCTGGTGCGCG |
| Homology región A Δ*hppD* | Primer SBF-UNS7-hppD-regionA-fw | CAAGACGCTGGCTCTGACATTTCCGCTACTGAACTACTCGGAGCCACTCCTTCTTCAGGA |
| | Primer SBF-UNS8-hppD-regionA-rev | CCAGGTGGTTGATGGGTTGATTGCTTTGGTTGAGACGAGGACTTCAAGGCCCTCTTCGAG |
| Homology región B Δ*hppD* | Primer SBF-UNS8-hppD-regionB-fw | CCTCGTCTCAACCAAAGCAATCAACCCATCAACCACCTGGGAGTAGGCGACGAGCTTCAT |
| | Primer SBF-UNS6-hppD-regionB-rev | TATGTGACCGTAGAGTATTCTTAGGTGGCAGCGAACGAGTGCGAGAGGTTCTTGATGC |
| Recombination checking Δ*hppD* | Recombination hppD FW | CGCTGCACCTCGACGCCG |
| | UNS8 REV | CCAGGTGGTTGATGGGTTG |
| Deletion checking Δ*hppD* | Deletion hppD fw | GTCGGCTTGTCCTGGACCG |
| | Deletion hppD rev | GGACCGGAGAACGGCAGC |
| Protospacer Δ*hppD* | Protospacer XNR_1801 fw | ACGCGGCCACGTGGCTCTCCAGGA |
| | Protospacer XNR_1801 rev | AAACTCCTGGAGAGCCACGTGGCC |
| pSEVA88c1 amplification | Vector FW UNS6 | CTCGTTCGCTGCCACCTAAGAATACTCTACGGTCACATACAAGCTTGCGGCCGCGTCG |
| | Vector REV UNS7 | CGAGTAGTTCAGTAGCGGAAATGTCAGAGCCAGCGTCTTGCCTAGGCGGCCTCCTGTG |
